# Job Burnout Among Primary Healthcare Workers in Rural China: A Multilevel Analysis

**DOI:** 10.3390/ijerph17030727

**Published:** 2020-01-22

**Authors:** Wanchun Xu, Zijing Pan, Zhong Li, Shan Lu, Liang Zhang

**Affiliations:** 1 School of Medicine and Health Management, Huazhong University of Science and Technology, Wuhan 430030, China; wanchunxu6@gmail.com (W.X.); peterpanzijing@gmail.com (Z.P.); lizhong@hust.edu.cn (Z.L.); shanlu@hust.edu.cn (S.L.); 2 Research Centre for Rural Health Service, Key Research Institute of Humanities & Social Sciences of Hubei Provincial Department of Education, Wuhan 430000, China

**Keywords:** burnout, rural health workforce, primary care, multilevel analysis

## Abstract

The health workers in rural primary care systems are at the increasing risk of job burnout. To explore the prevalence and associated factors of the job burnout among the primary healthcare worker in rural China, a cross-sectional survey was conducted among 15,627 participants in 459 township hospitals from six provinces. A combination of stratified multi-stage sampling and cluster sampling method, and a self-administrated questionnaire with the Chinese version of the Maslach Burnout Inventory-General Scale (MBI-GS) were used in the investigation. Multilevel regression analyses were used to examine the potential associated factors on both individual and organisational levels. 47.6% of respondents were experiencing moderate burnout, and 3.3% were in severe burnout. Professionals working for over 40 h per week, at young age, with a college degree, and with professional titles at medium or high rank reported a higher degree of job burnout. At the institutional level, the high ratio of performance-based salary was associated with a higher level of depersonalization. Burnout has become prevalent among the primary healthcare workers in rural China, and multiple strategies are needed to reduce the work stress and some high-risk groups’ vulnerability to job burnout.

## 1. Introduction

Job burnout is a significant problem among health workers [1,2]. Apart from its negative effect on the health of the medical staff [3], job burnout may also impair the productivity, responsiveness and availability of the health workforce. Job burnout was proved to be related to the lower job commitment [4], the higher turnover intention [5,6], as well as the poorer work performance of the health professionals [7,8,9,10]. The understandings of job burnout among the health workers would help to improve the management of the health workforce, which would also benefit the health system [11].

The burnout issue of health care practitioners has been widely studied thought out the USA and Europe in the past few decades, as well as in other developing areas recently [12]. The health workers in China were also experiencing the increased workload due to the soaring demand for health service and the shortage of health workforce. The number of physicians in China increased by 60% in the past two decades, but the number of patient visits and the inpatient admissions increased by 276% and 355% in the meantime [13]. It was reported in the previous studies that the prevalence of burnout among doctors in China ranged from 66.5% to 87.8%; young doctors and doctors working in general hospitals are found to be at higher risk of burnout [2]. The increasing demand for health care and weak primary healthcare system were considered to be the main driving factors of the rising burnout issue of the health workers in the general hospitals [14]. The majority of the research focus more on health professionals in general hospitals rather than those in the primary care system [2].

However, health workers in the primary health system were also confronted with increasing job stress in some developing countries, due to the shortage of health workforce [15] and increasing responsibilities demanded in the health reform [16,17]. Moreover, compared to the health professionals in the general hospitals, the health workers in the primary healthcare system are facing relatively less work resource, lower wages and less career development opportunities, especially in rural areas [15], which may also increase their perceived job stress [14,18]. Previous studies revealed that the majority of rural health workers are experiencing moderate burnout, and the common predictors included education level, years of employment, workload, and so on [14,18,19,20]. In China, the expanding duties of public health service provision [21], the new challenges merged from the health system reform [22,23,24], altogether with the long-lasting shortage of health workforce [25,26] are increasing the risks of job burnout among the health workers in the rural primary care system.

It has been proved that job burnout is related to the poorer work performance [27] and the higher turnover intention [18] of the health workers in the rural areas, which are also the main problems of the rural health workforce in current China [28,29]. The quality of primary care in rural areas is generally not satisfying, partly due to the work performance of the rural medical staff [30].It was reported that 85.8% of the outflow staff of the township hospitals were voluntary turnover [31].The high turnover rate of the health worker was also considered to be one of the main reasons for the lower efficiency and less satisfying performance of the primary care system in China [30,31]. Job burnout was an essential phenomenon in human resource management, which was also a potential antecedent of the problems mentioned above [32]. However, the existing researches on job burnout among the health workers in the rural primary care system of China, were still very limited in the volume and in geographic scope [18,20,27], which significantly limited the generalizability of the findings of the research. Moreover, according to the related theory and empirical evidence, the antecedents of burnout are usually divided into organisational, occupational and personal categories [32]. Most of the previous studies emphasised more on the factors on the individual level (occupational and personal categories). As most of the intervention strategies were designed and implemented on the institutional level, it is necessary to analyse the variances of burnout degree among different institutions and explore the potential determinants on the organisational level, such as the size of the institution, the work support, the reward system and other related factors [14,33,34].

To address these knowledge gaps, the present study aimed to investigate the prevalence and factors associated with job burnout among the primary healthcare workers in rural China. The geographically representative data on the national level, with the information of both individual and institutional characteristics, would be collected to gain better generalizability and interpretation of the study. The conclusions of the present study could help to develop the intervention strategies and improve the working condition for the rural health workers, which would also benefit the performance of the healthcare system in rural China.

## 2. Materials and Methods

### 2.1. Study Sample and Data Collection

A combination of stratified multi-stage sampling and cluster sampling method was applied in this cross-sectional survey. In the first stage, six provincial regions were selected based on the socio-economic level and geographic distribution (Eastern China: Shandong, Guangdong; Central China: Henan, Hubei; Western China: Guizhou and Chongqing). In the second stage, one developed city and one less-developed city (based on the gross regional product (GRP) per capita) were randomly selected in each provincial region. In the third stage, one district and two counties were randomly selected in each city. In Guangdong province, two districts in the developed city (Shenzhen) and four counties in the less-developed city (Shaoguan) were sampled in the survey. Apart from the 36 districts/counties initially sampled, another 6 counties in Shaoguan City also participated because the local health bureau asked to survey all of the primary care facilities. In total, 42 districts/counties were included in the present study. All the primary care institutions in the selected districts/counties were surveyed. At last, all health workers in-service in the sampled primary care facilities were invited to participate in the survey. In China, the majority of the township hospitals are located in the counties, while there are a small number of the township hospitals in the suburbs of the districts. The data from all the township hospitals sampled in each selected district/county, which served as the primary care facilities in rural areas [30], were included in the analysis in the present study.

The data were collected through an online survey in collaboration with the local health bureau in each selected county from October to November in 2018. The local health bureaus helped to initiate the contact between the study team and the local township hospitals online, and the leader of each township hospital was responsible for forwarding an online questionnaire consisting of demographic information, occupational information and measures of burnout in our name. All the participants were given consent to participate and assured de-identification and confidentiality in handling their data before they answer the questionnaires. After they finished the questionnaires online, all the survey data were directly delivered to our team without any intermediaries. Organisational information was also collected through an online questionnaire fulfilled by the administrators who were accessible to the related information in the township hospitals. The number of staff in-service in each township hospital was also recorded to estimate the response rate. The institutions that had not answered some of the variables in the present study were excluded from the analysis. In total, 16,404 respondents in 459 township hospitals participated in the survey, yielding a response rate of 86.4% (16,404 of 18,981). Cases with missing values on some items in the questionnaires were excluded from the analyses, and the final sample size was 15,627 with an effective response rate of 82.3%.

This study was approved by the Ethics Committee of Tongji Medical College, Huazhong University of Science and Technology (IORG No: IORG0003571).

### 2.2. Measures

#### 2.2.1. Burnout

The definition and measure that were most widely used in burnout research is the three-component model developed by Maslach and Jackson [1]. They defined burnout as a psychological syndrome of emotional exhaustion, depersonalization, and reduced personal accomplishment, which can occur among individuals who work with other people [1]. Validated by the extensive research in the past 30 years, the Maslach Burnout Inventory (MBI) is considered as the leading measure of burnout. MBI-General Survey was one of the versions of MBI and can be used in any occupational context [35] and was introduced into China in 2002 [36], which was used as the measuring instrument of job burnout in the present study.

The 15-item Chinese version of the Maslach Burnout Inventory-General Scale (MBI-GS) has shown good reliability and validity in the previous studies in China [7,28]. MBI-GS measures three dimensions of job burnout: (1) emotional exhaustion (5 items), feelings of being emotionally overextended and depleted of one’s emotional resource; (2) depersonalization (4 items), a negative, callous or excessively detached response to other people; (3) reduced personal accomplishment (6 items), a decline in one’s feelings of competence and successful achievement in one’s work [1]. Each item consists of a 7-point Likert scale ranging from 0 (“never”) to 6 (“every day”). Higher scores on the dimensions of emotional exhaustion and depersonalization indicate burnout, so as the lower scores on the dimension of personal accomplishment. The result of reliability analysis showed the scale was in a high level of internal consistency in all three dimensions in the current sample (Cronbach’s α = 0.943 for emotional exhaustion, α = 0.832 for depersonalization, and α = 0.882 for personal accomplishment subscales). The following equation was used to produce the weighted sum score of burnout, with the cut-off points for the classification, which was adopted by a number of previous studies [37,38]. The scores of six items of reduced personal accomplishment were reversed:

*Burnout = 0.4·exhaustion + 0.3·depersonalization + 0.3·reduced personal accomplishment*

According to the sum score of burnout, the participant can be divided into three groups, including group 1-no burnout (0–1.49), group 2-moderate burnout (1.50–3.49), and group 3-severe burnout (3.5–6.0) [37,38]. Participants with moderate or severe burnout were defined as a “burnout case” [10].

#### 2.2.2. Predictors

The predictors in the present study can be divided into three categories, including organisational, occupational and personal characteristics. In the context of the healthcare system in rural China, variables in each category were selected as below.

##### Personal Characteristics and Occupational Characteristics

Individual questionnaires were used to collect data on personal characteristics and occupational characteristics. We collected some key demographic information of the respondents, including gender (male/female), education level (high school or below/undergraduates and junior college students/postgraduates), age, marital status (married/unmarried). The unmarried category included single, widowed, divorced and separated status in the questionnaires. As for the occupational characteristics, we collected data on professional status (doctors/nurse/pharmacist/others), type of employment (temporary/long-term employee), years in profession, job tenure, the rank of professional title (primary/medium/high), administrative responsibility (yes/no) and workload (<40/≥40 h per week). The laboratorians and medical imaging specialists were also included in the category of doctors. Temporary employees included the staff hired as casuals or those rehired after retirement, which served as the important supplement of the workforce in the rural health system. Administrative responsibility referred to the directors of township hospitals and the departments.

##### Organisational Characteristics

We collected data on the type of institution (central/general township hospital), participation in the county medical alliance (yes/no) and the ratio of performance-based salary through the questionnaires on the institutional level. The detailed definitions were as followed. Firstly, Central township hospital was the officially-recognized medical centre in a certain region, which provided not only health services for the residents but also the technical supervision for the nearby general township hospitals. Generally, the size of the central township hospital was larger than that of the general township hospitals, so as more technically demanding services [39]. Secondly, the Chinese government is developing more and more county medical alliances for better health service delivery in the rural area, and some pilots have been taken place in some selected regions [40]. The township hospitals participating in the county medical alliances would gain more technical support and shared resources from the county hospital in the alliances. Thirdly, according to the performance-based salary policy for the health workers in the primary care system, the ratio of performance-based salary was recommended to be 30–40% in the national guideline and up to 70% in some regions [24,41]. This ratio was also surveyed in each township hospital.

### 2.3. Statistical Analysis

Cronbach’s α reliability coefficients for each dimension of burnout was calculated in SPSS version 24 (IBM Corp., Armonk, NY, USA). The estimation of the descriptive statistics of all measures, and the bivariate analysis of burnout scores with regard to individual-level predictors were conducted in Stata version 14 (StataCorp., College Station, TX, USA). All the continuous variables were transformed into the categorical variables in the analysis.

Multilevel regression analysis was conducted in Mplus Version 7 (Muthén & Muthén, Los Angeles, CA, USA) to investigate the association between burnout and the predictors mentioned above. The dependent variables included the sum scores in each dimension and weighted sum score of burnout. Each categorical variable was dummy-coded and tested against a reference group. Three nested models were generated for each dependent variable step by step. The first model (M0) is the null model that indicates the amount of variance in the dependent variable which is explained by second-level clusters (the institutions) to test if the multi-level analysis is justified for the current sample. The interclass correlation coefficient (ICC) is computed for each null model. Next, level one predictors (personal and occupational factors) entered the model and a distinct intercept is calculated for each institution (M1). In the third model, level two variables (organisational factors) entered to construct a two-level random intercept linear regression model (M2), of which the intercept of level one variables is predicted as a random effect of level two variables.

## 3. Results

### 3.1. General Characteristics of Sample

In total, 15,627 individuals in 459 township hospitals were included in the analysis. The characteristics of the subjects are detailed in Table 1. 33.6% of the sample institutions were central township hospitals (28.9% in the data of China Health Statistics Annual in 2017 [42]), and 68.4% have already participated in the county medical alliance in the local areas. 24.0% of the respondents were in the professional title of the medium or high rank (27.7% in the national annual [42]).

### 3.2. Prevalence of Burnout

The prevalence of burnout among the respondents was presented in Table 2. The proportion of the burnout cases were 50.9%, among whom 47.6% experienced moderate burnout while the remaining 3.3% suffered from severe burnout. The mean score of burnout score among the respondents was 1.59, and the median was 1.53 (Table 3), which met the criterion of moderate burnout. The comparison among different dimensions of burnout indicated that the degree of emotional exhaustion (median: 9; range: [0, 30]) is relatively higher than depersonalization (median: 3; range: [0, 24]) and the reduce personal accomplishment (median: 27; range: [0, 36], before reverse) in the current sample.

### 3.3. Factors Related to Burnout

The majority of the personal and occupational predictors were significant in the bivariate analysis (Table 4 and Table 5). he interclass correlation coefficient (ICC) in the null model for each dependent variable were 0.115 (emotional exhaustion), 0.088 (depersonalization), 0.071 (personal accomplishment) and 0.121 (sum scores of burnout), indicating that a multilevel analysis is necessary for each dependent variable (ICC > 0.059) [43]. 

In order to reduce the multicollinearity in the model, the predictors of years in profession and job tenure did not enter the model since they are highly correlated to the predictor of age group. The results of the multilevel regression analysis are provided in Table 6.

#### 3.3.1. Personal Characteristics

The professionals at the youngest group were experiencing a higher level of job burnout than those aged no less than 40 years old, as well as a lower level of personal accomplishment than all the other age groups (*p* < 0.001). Health workers with a college degree were in a higher degree of burnout (*p* = 0.003), as well as higher emotional exhaustion (*p* < 0.001) and depersonalization (*p* = 0.029). Nevertheless, the college degree also predicted a higher level of personal accomplishment (*p* = 0.016). The male workers were at a higher level of burnout than the female workers (*p* < 0.001). Married status predicted a lower level of burnout (*p* = 0.008).

#### 3.3.2. Occupational Characteristics

The workload of no less than 40 h per week was associated with a higher level of job burnout (*p* = 0.030) and emotional exhaustion (*p* < 0.001) as expected, but with a higher degree of personal accomplishment (*p* < 0.001). Doctors reported higher sum scores of burnout than nurses (*p* = 0.028), pharmacists (*p* < 0.001) and other technicians (*p* < 0.001). Health workers with the professional titles in medium and high rank were also in a higher level of burnout (*p* = 0.006) and emotional exhaustion (*p* < 0.001). The staff with administrative responsibility and the temporary staff reported a lower degree of burnout (*p* < 0.001).

#### 3.3.3. Organisational Characteristics

In order to investigate the effect of the low incentive policy and high incentive policy, the institutions with medium ratio (20–39%) of performance-based salary were set as the reference group. The high ratio (≥40%) of performance-based salary was found to be associated with a higher degree of depersonalization at the institutional level (*p* < 0.1). However, no difference was found between the institutions with a low ratio (0–19%) of incentive salary and the reference group. Type of institution and the participation in the county medical alliance were not significant predictors in the models for all three dimension and the sum scores of burnout.

## 4. Discussion

### 4.1. Prevalence of Burnout among Rural Health Workers

The present study demonstrated that 47.6% of the primary health care workers in rural China were in moderate burnout and 3.3% in severe burnout. The findings are consistent with the conclusions in some of the previous small-scale studies conducted in township hospitals in China [18,20,27,45]. 28.8% of the rural health workers in South Khorasan reported moderate burnout and 5.7% were in severe burnout [19], and these two proportions were 15.5% and 1.1% respectively among rural primary healthcare workers in Iran [17]. It should be noticed that the degree of job burnout among the rural primary healthcare workers in China was much higher. According to a national-scale monitoring data in China in 2011, 39.4% of the physicians in the township hospitals reported moderate burnout, and 0.3% were in severe burnout [46]. Compared to the result of our study, we can infer that the past few years have witnessed an increase of job burnout among the health workers in the township hospitals. It was possible that the increase of the patient visits in the township hospitals (about 20% from 2010 to 2017) [42], as well as the expanding duties and new challenges merged from the health system reform [14,18,22], had increased the workload of the rural primary care providers with a long-lasting shortage of health workforce [25,26], which might lead to the increase of job burnout Among the three dimensions of burnout, the respondents suffered most from emotional exhaustion, with reduced personal accomplishment followed, and least from depersonalization, which was also similar to the previous studies [18,45]. According to the theory of job burnout, emotional exhaustion was the initial stage [32], which implied that the burnout among the majority of the rural health workers was still at the early stage. The lower degree of depersonalization indicated the harmonious relationship between the rural health workers and their colleagues as well as the patients, which may be accounted for the cultural norm of the traditional society of acquaintances in rural China [45]. The lower personal accomplishment was reported and attached great importance to in many previous studies in the context of the primary care system in China. Lack of work autonomy due to restriction on the resources [47,48], as well as the patients’ distrust of the rural primary care institution [14], may contribute to the reduced personal accomplishment of some rural health workers.

However, there has not been much consensus on the “diagnosing” of burnout among healthcare professionals [49], and different version of MBI scales as well as various cut-off value system were applied in different studies.The comparison of the result among different studies and the interpretation should be cautious [50]. Moreover, there has not been validated cut-off value sets based on the normative sample for Chinese health professionals, and the development of such reference criterion may contribute to the validation of the increasing researches on job burnout among Chinese health professionals.

### 4.2. Factors Associated with Job Burnout

Some of the predictors in the present study were context-specific in the healthcare system in rural China, but the discussion on these factors would follow the classical theoretical frameworks of job burnout. One of the classic models is the Job Demands-Resources (JD-R) model, which considered that burnout arises when individuals experience incessant job demands and have inadequate resources available to address those demands [51]. Another famous model of job burnout is the Areas of Worklife (AW) model, which frames job stressors in terms of person-job imbalances and identifies six key areas in which these imbalances take place, including workload, control, reward, community, fairness and values [52].

Similar to previous studies, young professionals in the township hospitals are in a higher degree of job burnout. The lack of resource is wildly proved to be related to depersonalization and reduce personal efficacy [32]. The younger professionals generally had less work experience to copy with demanding situation [53]. The result in the bivariate analysis also indicated that as the years in profession and job tenure increased, the degree of burnout declined. Besides, in the hierarchy system in healthcare institutions [12], the younger staff have more time commitment [53] for the simple and repetitive tasks but less work autonomy [52], which may also lead to the reduced personal accomplishment. Moreover, the reward and career advancement opportunities for the young personals in rural areas were less than that in the urban area [14,15,54], increasing their vulnerability to job stress and burnout [52].

Due to the complicated history of the evolution of the medical academic degree system in China [55], there are a large proportion of primary health workers holding a high school or below education degree in rural China (21.1% in the present study). The health workers with college degrees reported a significantly higher degree of burnout. A similar result was also found in some previous studies [50], especially in the settings of the primary care system [14,19,20]. Maslach etc. suggested that the higher prevalence of job burnout among the highly educated people might be due to the higher responsibilities and higher stress, which helped to explain the higher personal accomplishment and higher emotional exhaustion respectively [56]. The health workers with higher education level also held higher job expectations [56]. When their expectations for their job were not met, they might be more likely to have job dissatisfaction [57], which could also lead to job burnout [19].

The type of employment was also worthy of attention. The continuing transformation of the Chinese economic system, along with the shortage of health workforce, had led to the evolution of the employment system in the healthcare system [58]. The proportion of the employees with “bianzhi” (a type of position with state-guaranteed lifetime employment) is decreasing, while the proportion of contract-based employees, which was so-called as “temporary staff” in the present study, is increasing [58]. The present study demonstrated a lower degree of burnout among the temporary staff. However, this predictor was silent in most previous studies [14,53]. The different situation may be due to the policy of improving the reward system for the temporary health workers in recent years, including the implementation of the “equal pay for equal work” policy [59], and more career advancement opportunities. For example, more “bianzhi” positions were allocated for the township hospitals to retain the rural health workforce and the temporary staff can gain the increased “bianzhi” through competition or examination [60,61,62,63], which may help to initiate the work enthusiasm and reduce the perceived job burnout.

As for other risk factors at the individual level, higher workload predicted a higher level of job burnout as expected [2,14]. It should be noticed that nearly all of the rural health workers (93.1%) reported working for over 40 h per week, which was much higher than that reported in the community care centres in some urban area (25.8–46.8%) [64]. The workers with professional title in the medium (20.5%) or high (3.5%) rank were experiencing a higher level of job burnout. In the Chinses healthcare system, most of the professionals with medium titles were in the promotion period of career, during which the heavy workload, competition as well as enterprising ambition might result in the high level of burnout [65]. The married status predicted a lower degree of burnout, while the male workers reported a higher level of job burnout at the present study. The deviation from some previous studies [65,66] may be mainly due to the different culture and social norms in different settings among the studies. Therefore, the present study also expanded the knowledge of the associated factors of job burnout in the context of rural primary care system of China.

The ICC of the null models indicated that the level of burnout varied among the institutions, which suggested that the organisational environment also played an essential role in the job burnout in the rural primary healthcare system. Only the ratio of performance-based salary was significant in the model for the dimension of depersonalization. It has been widely proved that the reward system was critical to the management of job burnout [52]. China has launched the reform of performance-based salary in primary healthcare institutions since 2009 [41]. Previous evidence demonstrated that this strategy was not a strong incentive in most of the areas, and the main reason was the inadequate individual total salary of the primary care providers [41]. Our study also found that there was no significant difference of the burnout level between the township hospitals with lower ratio of performance-based salary (0–19%) and those at medium ratio (20–39%). However, our study found the township hospitals with higher ratio of performance-based salary were experiencing a higher level of depersonalization than those at the medium ratio. It was considered that the over-emphasizing on the individual incentive might impair the teamwork and internal relationship among the workers [67], so the excessive competition among the staff may influence the colleague relationship, which may result in the high level of the depersonalization. Besides, it had been proved that the employees’ burnout and job satisfaction were related to the employees’ perceptions of performance appraisal, such as the instrument validity, distributive justice and the procedural justice [34]. The underdeveloped performance appraisal systems for the staff in the township hospitals was found to be related to the health workers’ dissatisfaction of the performance-based salary in some regions. [41,68]. The dissatisfaction in the reward system could increase the employees’ vulnerability to job burnout [52].

No significant difference of the burnout level was found between the central township hospitals and general township hospitals. Although the central township hospitals took more responsibilities in the health system in the local region, the reward and work resources could also be higher in the central township hospitals than those in the general township hospitals [69], which could decrease the workers’ vulnerabilities to job burnout [52]. The county medical alliances were expected to influence the job burnout level of rural health workers. One of the capacity-building strategies of county medical alliances was the sharing of the medical resources and technical support provided by the county hospitals for the primary care institutions [70]. According to the JD-R model of burnout, the increase in the work resources and support may help to facilitate the work in township hospitals, and consequently reduced their job burnout level. However, this predictor was not significant in the models of the present study. The influence of the county medical alliance policy on the health workforce in rural primary care institutions remained to be studied.

### 4.3. Implications

The result of the present study highlighted the significance of the burnout issue among primary healthcare workers in rural China. Confronted with the increasing responsibilities and the long-lasting shortage of health workforce, the workload of the rural primary health care workers has been increasing in the past few years. Apart from the on-going policy of the health workforce retention and development to relieve the shortage of workers [71], some duties and demands merged from the reform of the health system were also called to be improved or simplified to reduce the excessive workload, such as the assessment standards of National Basic Public Health Service Program [14].

We identified some groups of high-risk population of job burnout, including the young professionals, workers with the higher education degree and higher professional titles. Integrated strategies were recommended to strengthen the work support for the young rural health workers to facilitate their adaption in the working place and thus reduce their experience of job burnout. Besides, both the adequate material rewards and other measures to improve the intrinsic satisfaction, such as more career advancement opportunities, are necessary to fulfil the high work expectation of these populations [32,52].

The use of multilevel analysis also provided new insight into the organisational factors. The result suggested that the organisational environment did matter. However, the existing researches on the organisational factors related to job burnout are limited in volume. Since these factors are more changeable and valuable in the design of organisational or administrative policies, further studies were needed to discover the organisational determinants of job burnout in different contexts. The present study found that the effectiveness of the policy of performance-based salary was limited, and the over-incentive strategies would also lead to a higher degree of depersonalisation among the staff, implying that the internal relationships may be impaired by the over-competition. Apart from raising the salary to improve the effectiveness of the policy [41], a shift from the overemphasise on the individual performance to the team-based incentives [67,72] is also worthy of attention in the design of the organisational incentive systems in future.

### 4.4. Limitations

There may be a selection bias in the present study since not all of the stuff in each sampled institution participated in the survey. However, the high response rate of 86.4% helped to minimise the influence of this selection bias. Another important limitation was the heterogeneous structure of the sample group. The nature of the work, as well as the associated factors of job burnout, were different among the health workers in different professions, which were not analysed and discussed separately in the present study. Further studies were needed to illustrate the situation in different professions to gain insights for the specific and differential strategies for burnout control. Moreover, the cross-sectional design of the present study limited our ability to draw causal inferences. No causal conclusions can be established in the present study.

## 5. Conclusions

The present study revealed the prevalence and associated factors of job burnout among the primary healthcare workers in rural China, based on the geographically representative survey data across China and the standard measuring instrument. Nearly half of the respondents were experiencing job burnout. Several high-risk groups were identified, such as the young professionals, professionals with higher education degree and higher rank of professional title, and the long-term employees. The multilevel analysis demonstrated that the organisational environment played an important role in the job burnout, such as the incentive policy of the performance-based salary. The findings of the present study highlight the importance of several aspects for the intervention of job burnout, such as the allocation of more health workers, the improvement in the assessment system and the design of the reward system-. The control of the job burnout is necessary for the well-being of the rural primary healthcare workers, which would also benefit the performance of the healthcare system.

## Figures and Tables

**Table 1:** General characteristics of the sampled participants (*n* = 15,627) and institutions (*n* = 459).

Individual		*N*	%
Age group (years)			
	≤29	5391	34.5
	30–39	5173	33.1
	40–49	3889	24.9
	≥50	1174	7.5
Gender			
	Male	5289	33.8
	Female	10,338	66.2
Marital status			
	Married	12,319	78.8
	Unmarried	3308	21.2
Education level			
	High school or below	3304	21.1
	Undergraduates *	12,310	78.8
	Postgraduates	13	0.1
Years in profession			
	0–9	6998	44.8
	10–19	4044	25.9
	20–29	3503	22.4
	≥30	1082	6.9
Job tenure (years)			
	0–9	9468	60.6
	10–19	2994	19.2
	20–29	2424	15.5
	≥30	741	4.7
Professional title			
	Primary or below	11,876	76.0
	Medium	3206	20.5
	High	545	3.5
Professional status			
	Physicians	8898	56.9
	Nurse	4419	28.3
	Pharmacist	1200	7.7
	Others	1110	7.1
Administrative responsibility			
	Yes	5631	36.0
	No	9996	64.0
Type of employment			
	Temporary	3557	22.8
	Long-Term	12,070	77.2
Workload (hours per week)			
	<40	1073	6.9
	≥40	14,554	93.1
**Institution**			
Type of institution			
	Central	154	33.6
	General	305	66.4
County medical alliance			
	Yes	314	68.4
	No	145	31.6
Ratio of performance-based salary			
	0–19%	72	15.7
	20–39%	201	43.8
	≥40%	186	40.5

Note: * Undergraduates: the junior college students were also included in this category.

**Table 2:** Prevalence of job burnout among the workers in rural primary care institutions (*n* = 15627).

Degree	Range	N	%
No burnout symptoms	0–1.49	7664	49.0
Some burnout symptoms	1.50–3.49	7443	47.6
Serious burnout symptoms	3.50–3.49	520	3.3

**Table 3:** Scores in each dimension and the weighted sum score of job burnout.

	Score	
	Mean ± SD	Median (Range)
Emotional exhaustion	9.84 ± 6.57	9.0 (0,30.0)
Depersonalization	4.12 ± 4.40	3.0 (0,24.0)
Personal accomplishment ^1^	26.05 ± 8.33	27.0 (0,36.0)
Weighted sum score of burnout	1.59 ± 0.96	1.53 (0,5.84)

^1^ Note: the scores of items of personal accomplishment were not reversed in the analysis of single dimension.

**Table 4:** The scores of burnout by personal characteristics and the result of bivariate analysis.

	Burnout		Emotional Exhaustion		Depersonalization		Personal Accomplishment	
	Mean (SD)	Median (Range)	Mean (SD)	Median (Range)	Mean (SD)	Median (Range)	Mean (SD)	Median (Range)
Age group (years)								
≤29	1.64 (0.96)	1.60 (0,5.84)	9.10 (6.15)	9.00 (0,30.00)	4.36 (4.40)	4.00 (0,24.00)	24.42 (8.58)	25.00 (0,36.00)
30–39	1.64 (0.98)	1.57 (0,5.70)	10.27 (6.70)	10.00 (0,30.00)	4.36 (4.54)	3.00 (0,24.00)	26.06 (8.24)	27.00 (0,36.00)
40–49	1.52 (0.93)	1.42 (0,5.35)	9.98 (6.66)	9.00 (0,30.00)	3.70 (4.24)	2.00 (0,24.00)	27.39 (7.91)	30.00 (0,36.00)
≥50	1.43 (0.95)	1.32 (0,5.13) **	9.51 (7.39)	8.50 (0,30.00) **	3.38 (4.17)	2.00 (0,24.00) **	27.67 (8.30)	30.00 (0,36.00) **
Gender								
Male	1.63 (1.00)	1.56 (0,5.70)	10.35 (7.27)	10.00 (0,30.00)	4.16 (4.69)	3.00 (0,24.00)	26.32 (8.27)	28.00 (0,36.00)
Female	1.58 (0.94)	1.51 (0,5.84) *	9.42 (6.17)	9.00 (0,30.00) **	4.10 (4.26)	3.00 (0,24.00) *	25.75 (8.43)	27.00 (0,36.00) **
Marital status								
Married	1.57 (0.95)	1.49 (0,5.70)	9.85 (6.61)	9.00 (0,30.00)	3.99 (4.34)	3.00 (0,24.00)	26.45 (8.24)	28.00 (0,36.00)
Unmarried	1.69 (0.99)	1.67 (0,5.84) **	9.29 (6.42)	9.00 (0,30.00) **	4.60 (4.60)	4.00 (0,24.00) **	24.08 (8.63)	24.00 (0,36.00) **
Education level								
High school or below	1.44 (0.95)	1.33 (0,5.54)	8.57 (6.72)	8.00 (0,30.00)	3.54 (4.24)	2.00 (0,24.00)	26.32 (8.77)	29.00 (0,36.00)
Undergraduates and junior college	1.63 (0.96)	1.57 (0,5.84)	10.05 (6.50)	9.00 (0,30.00)	4.27 (4.44)	3.00 (0,24.00)	25.85 (8.27)	27.00 (0,36.00)
Postgraduates	1.89 (1.33)	1.81 (0,5.03) **	10.23 (7.75)	10.00 (0,30.00) **	5.77 (5.70)	5.00 (0,19.00) **	23.15 (11.15)	24.00 (0,36.00) **

Note: the variables of emotional exhaustion (skewness = 0.84, kurtosis = 3.74), depersonalization (skewness = 1.47, kurtosis = 5.78), personal accomplishment (skewness = −0.44, kurtosis = 0.55) and the weighted sum scores of burnout (skewness = −0.55, kurtosis = 3.24) did not meet the criteria for normal univariate distribution (*p* < 0.01). Therefore, Kruskal-Wallis test was applied in the bivariate analysis, so as the result in Table 5. * *p* value < 0.01, ** *p* value < 0.001.

**Table 5:** The scores of burnout by occupational characteristics and the result of bivariate analysis.

	Burnout		Emotional Exhaustion		Depersonalization		Personal Accomplishment	
	Mean (SD)	Median (Range)	Mean (SD)	Median (Range)	Mean (SD)	Median (Range)	Mean (SD)	Median (Range)
Years in profession								
0–9	1.63 (0.96)	1.59 (0,5.84)	9.21 (6.22)	9.00 (0,30.00)	4.32 (4.38)	4.00 (0,24.00)	24.64 (8.56)	25.00 (0,36.00)
10–19	1.63 (0.97)	1.55 (0,5.70)	10.39 (6.75)	10.00 (0,30.00)	4.27 (4.53)	3.00 (0,24.00)	26.44 (8.13)	28.00 (0,36.00)
20–29	1.52 (0.94)	1.43 (0,5.18)	10.09 (6.69)	9.00 (0,30.00)	3.77 (4.32)	2.00 (0,24.00)	27.47 (7.91)	30.00 (0,36.00)
≥30	1.44 (0.96)	1.32 (0,5.33) **	9.51 (7.48)	8.00 (0,30.00) **	3.39 (4.25)	2.00 (0,24.00) **	27.62 (8.29)	30.00 (0,36.00) **
Job tenure (years)								
0–9	1.64 (0.97)	1.58 (0,5.84)	9.58 (6.39)	9.00 (0,30.00)	4.30 (4.43)	3.00 (0,24.00)	25.10 (8.47)	26.00 (0,36.00)
10–19	1.60 (0.95)	1.52 (0,5.63)	10.31 (6.85)	10.00 (0,30.00)	4.12 (4.43)	3.00 (0,24.00)	26.72 (8.12)	28.00 (0,36.00)
20–29	1.48 (0.95)	1.40 (0,4.88)	9.83 (6.70)	9.00 (0,30.00)	3.71 (4.34)	2.00 (0,24.00)	27.79 (7.85)	30.00 (0,36.00)
≥30	1.38 (0.92)	1.26 (0,5.13) **	9.10 (7.28)	8.00 (0,30.00) **	3.13 (4.08)	2.00 (0,24.00) **	27.60 (8.38)	30.00 (0,36.00) **
Professional status								
Physicians	1.64 (0.98)	1.59 (0,5.80)	10.23 (6.80)	10.00 (0,30.00)	4.25 (4.55)	3.00 (0,24.00)	25.89 (8.36)	27.00 (0,36.00)
Nurse	1.58 (0.93)	1.51 (0,5.84)	9.36 (6.08)	9.00 (0,30.00)	4.16 (4.21)	3.00 (0,24.00)	25.72 (8.45)	27.00 (0,36.00)
Pharmacist	1.42 (0.94)	1.30 (0,5.45)	8.58 (6.32)	8.00 (0,30.00)	3.57 (4.12)	2.00 (0,24.00)	26.76 (8.20)	28.00 (0,36.00)
Others	1.42 (0.95)	1.33 (0,5.54) **	8.48 (6.49)	8.00 (0,30.00) **	3.52 (4.27)	2.00 (0,24.00) **	26.46 (8.45) **	28.00 (0,36.00) **
Professional title								
Primary or below	1.60 (0.96)	1.54 (0,5.84)	9.50 (6.53)	9.00 (0,30.00)	4.15 (4.41)	3.00 (0,24.00)	25.42 (8.52)	26.00 (0,36.00)
Medium	1.58 (0.95)	1.49 (0,5.70)	10.55 (6.57)	10.00 (0,30.00)	4.12 (4.44)	3.00 (0,24.00)	27.53 (7.67)	30.00 (0,36.00)
High	1.45 (0.93)	1.4 (0,4.83) **	10.03 (7.17)	9.00 (0,30.00) **	3.39 (4.07)	2.00 (0,24.00) **	28.16 (7.76)	31.00 (0,36.00) **
Administrative responsibility								
Yes	1.54 (0.96)	1.46 (0,5.84)	9.92 (6.80)	9.00 (0,30.00)	3.82 (4.33)	3.00 (0,24.00)	26.81 (8.10)	29.00 (0,36.00)
No	1.62 (0.96)	1.56 (0,5.70) *	9.63 (6.44)	9.00 (0,30.00) *	4.29 (4.44)	3.00 (0,24.00) **	25.46 (8.49)	26.00 (0,36.00) **
Type of employment								
Temporary employee	1.50 (0.91)	1.42 (0,5.54)	8.43 (6.04)	8.00 (0,30.00)	3.75 (4.09)	3.00 (0,24.00)	25.26 (8.70)	26.00 (0,36.00)
Long-term employee	1.62 (0.98)	1.55 (0,5.84) *	10.12 (6.68)	9.00 (0,30.00) **	4.23 (4.49)	3.00 (0,24.00) **	26.15 (8.27)	27.00 (0,36.00) **
Workload (hours per week)								
<40	1.54 (0.96)	1.50 (0,5.45)	8.31 (6.46)	8.00 (0,30.00)	4.12 (4.40)	3.00 (0,24.00)	24.52 (8.87)	25.00 (0,36.00)
≥40	1.60 (0.96)	1.53 (0,5.84)	9.84 (6.57)	9.00 (0,30.00) **	4.09 (4.45)	3.00 (0,24.00)	26.05 (8.33)	27.00 (0,36.00) **

* *p* value < 0.01, ** *p* value < 0.001.

**Table 6:** The results of the multilevel regression analysis of the factors associated with burnout.

		Burnout		EE ^1^		DP ^2^		PA ^3^	
		M1	M2	M1	M2	M1	M2	M1	M2
	Constant	1.645 **	1.628 **	7.486 **	7.501 **	4.658 **	4.513 **	22.070 ***	22.221 **
Personal characteristics									
Age group	≤29 (ref ^4^)	−	−	−	−	−	−	−	−
	30–39	0.033	0.033	0.837 **	0.836 **	0.064	0.062	0.807 **	0.806 **
	40–49	−0.116 **	−0.116 **	0.388 **	0.389 **	−0.676 **	−0.675 **	1.927 **	1.927 **
	≥50	−0.178 **	−0.178 **	0.177	0.177	−0.932 **	−0.931 **	2.457 **	2.454 **
Gender	Female (ref)	−	−	−	−	−	−	−	−
	Male	0.103 **	0.103 **	0.844 **	0.843 **	0.379 **	0.378 **	−0.138	−0.139
Marital status	Unmarried (ref)	−	−	−	−	−	−	−	−
	Married	−0.064 **	−0.064 **	0.122	0.121	−0.379 **	−0.380 **	0.968 **	0.966 **
Education	Non-college	−	−	−	−	−	−	−	−
	College degree	0.062 **	0.062 **	0.911 **	0.914 **	0.199 **	0.200 **	0.459 **	0.461 **
Occupational characteristics									
Profession status	Physicians (ref)	−	−	−	−	−	−	−	−
	Nurse	−0.045 **	−0.045 **	−0.331 **	−0.332 **	−0.072	−0.072	0.265	0.263
	Pharmacist	−0.185 **	−0.185 **	−1.286 **	−1.286 **	−0.576 **	−0.574 **	0.813 **	0.808 **
	Others	−0.149 **	−0.149 **	−1.126 **	−1.124 **	−0.449 **	−0.447 **	0.534 *	0.535 *
Professional title	Primary or below (ref)	−	−	−	−	−	−	−	−
	Medium and high	0.064 **	0.064 **	0.808 **	0.813 **	0.347 **	0.350 **	0.624 **	0.621 **
Administrative ^5^	No (ref)	−	−	−	−	−	−	−	−
	Yes	−0.075 **	−0.075 **	−0.167	−0.169	−0.437 **	−0.437 **	0.601	0.597
Employment	Long-term employee (ref)	−	−	−	−	−	−	−	−
	Temporary employee	−0.109 **	−0.109 **	−0.776 **	−0.776 **	−0.473 **	−0.470 **	0.224	0.218
Workload ^6^	<40 (ref)	−	−	−	−	−	−	−	−
	≥40	0.065 **	0.065 **	1.369 **	1.370 **	0.110	0.109	1.080 **	1.086 **
Organizational characteristics									
Institution type	General (ref)	−	−	−	−	−	−	−	−
	Central	−	−0.037	−	−0.253	−	−0.183	−	0.066
Medical alliance	No (ref)	−-	−	−	−	−	−	−	−
	Yes	−	0.010	−	−0.125	−	0.097	−	−0.215
P-B salary ^7^	20–39% (ref)	−	−	−	−	−	−	−	−
	0–19%	−	0.004	−	0.037	−	0.147	−	0.129
	≥40%	−	0.056	−	0.388	−	0.296 *	−	−0.127

Note: Robust Maximum Likelihood Estimator (robust to non-normality data) was used to estimate the model parameters [33,44]. Abbreviation: (1) EE: emotional exhaustion; (2) DP: depersonalization; (3) PA: personal accomplishment; (4) ref: reference group; (5) administrative responsibility; (6) hours per week; (7) P-B salary: ratio of performance-based salary. * *p* value < 0. 1, **p value < 0.05.
